# Prevalence of Diagnosed Essential Tremor Using Electronic Healthcare Records: A Population-Based Study in Northeast-Central Pennsylvania

**DOI:** 10.5334/tohm.1229

**Published:** 2026-07-16

**Authors:** Daniel Kratovil, Seung Yeon Jung, Sonia Stoica, Matthew Roy, Alia C. Stanciu, M. Cosmin Sandulescu

**Affiliations:** 1 Department of Neurology, Geisinger Medical Center, Danville, PA, United States; 2 Geisinger Medical Center, Danville, PA, United States; 3 Freeman College of Management, Bucknell University, Lewisburg, PA, United States; 4 Geisinger Commonwealth School of Medicine, Scranton, PA, United States

**Keywords:** prevalence, diagnosed essential tremor, electronic healthcare records

## Abstract

**Background:**

Essential tremor (ET) is one of the most common movement disorders, yet reported prevalence estimates vary widely, likely because of differences in ascertainment strategies, which can influence data validity and comparability. Electronic healthcare records (EHRs) offer an opportunity to estimate prevalence at the population level. However, the impact of these strategies remains incompletely understood, raising questions about the accuracy and generalizability of the resulting estimates.

**Methods:**

We conducted a retrospective, population-based cross-sectional study using EHR data from the Geisinger Health System (GHS) to identify cases of ET. Cases were mapped to a census-defined population of 1,081,934 individuals aged ≥10 years in Northeast–Central Pennsylvania on April 1, 2020. ET prevalence was assessed using two ascertainment strategies: (1) the neurology cohort and (2) the all-specialties cohort. Age-threshold, age-specific, and sex-stratified prevalence estimates were calculated, along with age-standardized rates based on the 2020 U.S. population.

**Results:**

Crude prevalence of ET was 0.261% (95% CI, 0.252–0.271) in the neurology cohort and 0.653% (95% CI, 0.638–0.668) in the all-specialties cohort. Age-standardized prevalence was 0.40% (95% CI, 0.39–0.41) and 1.01% (95% CI, 1.00–1.02), respectively. Prevalence increased progressively with age across both ascertainment strategies, and sex-stratified analyses demonstrated similar age-related patterns with a modest female predominance, particularly in the all-specialties cohort.

**Discussion:**

ET prevalence estimates vary substantially depending on ascertainment strategy, with broader EHR-based definitions identifying more than twice as many cases as neurologist-restricted approaches. These findings highlight the importance of standardization in improving comparability across studies.

## Introduction

Essential tremor (ET) is increasingly recognized as a clinically heterogeneous disorder rather than a purely benign tremor syndrome. Although classically defined by upper-limb kinetic and postural tremor [1], accumulating evidence indicates that ET is associated with functional impairment, reduced quality of life, and non-motor features, including cognitive decline and mood disturbances [2,3,4,5,6]. In addition, longitudinal studies suggest a potential association between ET and an increased risk of neurodegenerative conditions, including Parkinson’s disease (PD) [4,7]. Despite its high prevalence worldwide [8], the epidemiology of ET remains incompletely understood, with substantial variability in prevalence estimates across studies. This variability is likely driven by differences in case definition, ascertainment strategies, and population characteristics. Improved understanding of ET prevalence within defined populations is therefore essential to enhance diagnostic recognition, inform healthcare planning, and better characterize the broader impact of this condition.

Reported prevalence estimates of ET in the United States vary widely, ranging from approximately 0.28 to 5.5% [9,10,11,12,13,14,15], depending on study design, population, and diagnostic criteria. Prevalence increases markedly with age, with higher estimates observed in older adults [15,16]. Importantly, studies based on clinical diagnosis or administrative data tend to report lower prevalence, whereas those incorporating direct neurologic examination and broader case definitions yield higher estimates. These differences underscore the central role of ascertainment methodology in shaping epidemiologic estimates of ET.

Northeast-Central Pennsylvania (NECP) presents a unique setting for epidemiological research on ET due to its demographic composition, environmental exposures, and healthcare accessibility factors. ET is frequently underdiagnosed or misdiagnosed, particularly in settings with limited access to movement disorder specialists [17]. Improved characterization of ET prevalence in this region may therefore support earlier diagnosis, more targeted resource allocation, and greater access to appropriate treatment and support services.

Given these gaps, the present study aims to determine the crude prevalence of diagnosed ET in NECP using a large population-based EHR dataset, and to evaluate how different ascertainment strategies affect these estimates. We hypothesized that ET prevalence is not a fixed measure but varies systematically across ascertainment strategies applied to the same population.

## Methods

### Study Design and Setting

This retrospective cross-sectional study examined the prevalence of diagnosed ET on a fixed date using EHR data from Geisinger Health System (GHS), the largest integrated healthcare network in NECP. GHS serves more than 40 counties through a network of hospitals, outpatient clinics, and specialty practices [18]. The 13 counties included in this analysis (Centre, Clinton, Columbia, Juniata, Lackawanna, Luzerne, Lycoming, Mifflin, Montour, Northumberland, Snyder, Union, and Wyoming) were selected a priori because they encompass the geographic region surrounding Geisinger’s major hospitals and outpatient facilities and represent its primary service area [19].

Residence within the study region was determined using the patient’s residential ZIP code recorded in the EHR at the time of data extraction. Patients whose ZIP code corresponded to one of the thirteen study counties were considered residents of the study region. Because historical residential information was unavailable, residence was assessed using the most recently recorded address rather than verified on the prevalence date. To ensure precise alignment between the numerator of identified ET cases and the denominator of the underlying population, the investigators anchored all analyses to April 1, 2020, the reference date of the 2020 United States Decennial Census. Age-specific and sex-specific population counts for these thirteen counties were obtained directly from the Census Demographic Profile [20].

The source population was restricted to individuals who were alive on the prevalence date (April 1, 2020) and had received an ET diagnosis on or before that date. Exclusion diagnoses were identified using all available EHR data through the time of data extraction in May 2024. Accordingly, individuals subsequently diagnosed with an exclusion condition after the prevalence date were excluded from the final ET case definition. Eligible cases were further restricted to patients who were 10 years of age or more by the time of their first documented ET diagnosis. Age was calculated as of April 1, 2020, and participants were grouped into standard 10-year age strata (10–19, 20–29, …, 70–79). In addition, we also calculated prevalence for successively higher minimum age-threshold groups (individuals 10 years or older, 20 years or older, …, 80 years or older) to examine age-dependent patterns.

Cases were categorized into three groups. The neurology cohort included individuals with at least one ET diagnosis assigned by a neurologist. The non-neurology cohort included individuals with ET diagnoses assigned exclusively by non-neurology providers and no recorded ET diagnosis from a neurologist. The all-specialties cohort included all individuals with an ET diagnosis regardless of provider specialty and therefore consisted of the combined neurology and non-neurology cohorts.

Prevalence estimates were calculated using two ascertainment strategies: (1) using the neurology cohort and (2) using the all-specialties cohort. In addition, age-at-diagnosis analyses were performed as a sensitivity analysis comparing the neurology and non-neurology cohorts to evaluate whether individuals who never received an ET diagnosis from a neurologist differed in age at diagnosis from those evaluated by neurologists.

To enhance phenotypic precision beyond reliance on administrative ICD codes alone, which may aggregate heterogeneous tremor syndromes under shared rubrics such as ICD-9 code 333.1, the study incorporated structured data elements from epic diagnosis groupers (EDG) [21] and documented electronic differential diagnoses (DDX) [22]. This contextual information enabled investigators to distinguish primary ET from alternative tremor etiologies more reliably. Furthermore, a rigorous set of exclusion criteria was applied to reduce misclassification. Patients were excluded if their records contained diagnostic codes indicating drug-induced tremor, secondary tremor syndromes, atypical parkinsonism, PD, cerebellar disorders (including spinocerebellar ataxias), or dystonia. This dual-strategy design was informed by prior work showing that neurologist-assigned ET diagnoses in the GHS EHR exhibit high sensitivity (94–96%) when adjudicated against detailed clinical chart review using both the 1998 and 2018 Movement Disorder Society consensus criteria [23]. Diagnostic confidence further increased when the diagnosis appeared across multiple neurology encounters, supporting the use of neurologist documentation as a robust proxy for clinically recognized ET in population-level research.

The second ascertainment strategy included all-specialties ET cases identified from the EHR regardless of provider specialty. Individuals were eligible if they had a documented ET diagnosis assigned by any healthcare provider within the GHS. To maintain consistency across ascertainment strategies, the same exclusion criteria applied to the neurology cohort were also applied to the all-specialties cohort, including exclusion of drug-induced tremor, secondary tremor syndromes, atypical parkinsonism, cerebellar disorders, dystonia, and PD. Unlike the neurology cohort, however, the additional non-neurology cases contributing to the all-specialties cohort were not subjected to formal chart validation because documentation from non-neurology providers frequently lacked sufficient clinical detail. This broader ascertainment strategy was intended to provide an upper-bound estimate of diagnosed ET prevalence within the healthcare system, while recognizing that some diagnostic misclassification may persist.

Age and sex-specific population counts derived from the 2020 U.S. Census served as the denominators for all prevalence calculations. Population counts were stratified using the same 10-year age strata and successively nested minimum-age threshold groups used for prevalence estimation, and were further disaggregated by sex for sex-specific analyses.

The primary outcome was the point prevalence of ET on April 1, 2020, expressed as a percentage. Crude, age-specific, age-threshold, and sex-stratified prevalence values were calculated for both ascertainment strategies. Direct age-standardization was performed using the 2020 U.S. standard population [24] to facilitate comparison between the neurology and all-specialties cohorts. All prevalence estimates were reported with 95% confidence intervals (CIs). CIs for the age-specific and age-threshold prevalence estimates were calculated assuming a binomial distribution, whereas CIs for age-standardized estimates were derived using a Poisson-based approximation.

Sex-stratified analyses were conducted in parallel, allowing direct comparison of patterns between males and females in regard to both age-specific and age-threshold prevalence. Sex differences were calculated as female prevalence minus male prevalence. Comparative analyses additionally examined differences in prevalence estimates produced by the two ascertainment strategies and assessed whether the age distribution at diagnosis differed between cohorts using non-parametric statistical methods.

All data extraction, cleaning, age categorization, prevalence estimation, and visualization procedures were executed in R (version 4.0.2, R Foundation for Statistical Computing, Vienna, Austria) [25].

The study was approved by the Geisinger Medical Center Institutional Review Board (2026–0545). Because the investigation utilized deidentified EHR data, the requirement for informed consent was waived.

## Results

The study population comprised 1,081,934 individuals aged 10 years or older residing in NECP, based on 2020 U.S. Census data [20]. The sex distribution was nearly equal overall, with 49.5% males and 50.5% females; however, a predominance of females was observed in older age groups, reflecting broader demographic trends. Population counts generally decreased across the older age groups, ranging from 172,472 individuals aged 20–29 years to 60,937 individuals aged 80 years or older.

Crude ET prevalence, as determined by EHR-based case ascertainment, varied considerably depending on diagnostic criteria and increased substantially with age (Table 1). Under the more restrictive neurology definition, the crude prevalence among individuals aged 10 years or older was 0.261% (95% CI 0.252–0.271), compared to 0.653% (95% CI 0.638–0.668) when cases identified through all-specialties ascertainment were included. The corresponding age-standardized prevalence estimates, based on the 2020 U.S. Census, were 0.40% (95% CI 0.39–0.41) and 1.01% (95% CI 1.00–1.02), respectively. Both ascertainment methods showed a pronounced age influence, with prevalence increasing from 0.027% (neurology) or 0.049% (all-specialties) in the 10–19-year age-specific group to 0.607% and 1.427%, respectively, among individuals aged 70–79 years. Prevalence for age-threshold group also increased with higher age thresholds, reaching 0.675% (neurology) and 1.683% (all-specialties) among those aged 70 years or older.

**Table 1:** Age-specific and Age-threshold Prevalence of Essential Tremor by Ascertainment Strategy.


AGE-SPECIFIC	POPULATION	NEUROLOGY			ALL-SPECIALTIES		
	
		CASES	PREVALENCE	95% CI	CASES	PREVALENCE	95% CI

**10–19**	145,721	40	0.027	0.020–0.037	71	0.049	0.038–0.061

**20–29**	172,472	156	0.090	0.077–0.106	366	0.212	0.191–0.235

**30–39**	142,935	190	0.133	0.115–0.153	566	0.396	0.364–0.430

**40–49**	135,245	229	0.169	0.148–0.193	684	0.506	0.469–0.545

**50–59**	161,620	466	0.288	0.263–0.316	1114	0.689	0.649–0.731

**60–69**	158,684	630	0.397	0.367–0.429	1482	0.934	0.887–0.983

**70–79**	104,320	633	0.607	0.561–0.656	1489	1.427	1.356–1.501

**AGE-THRESHOLD**		–	–	–	–	–	–

**10+**	1,081,934	2827	0.261	0.252–0.271	7065	0.653	0.638–0.668

**20+**	936,213	2787	0.298	0.287–0.309	6994	0.747	0.730–0.765

**30+**	763,741	2631	0.344	0.332–0.358	6628	0.868	0.847–0.889

**40+**	620,806	2441	0.393	0.378–0.409	6062	0.976	0.952–1.001

**50+**	485,561	2212	0.456	0.437–0.475	5378	1.108	1.079–1.137

**60+**	323,941	1746	0.539	0.514–0.565	4264	1.316	1.278–1.356

**70+**	165,257	1116	0.675	0.637–0.716	2782	1.683	1.623–1.747

**80+**	60,937	483	0.793	0.725–0.866	1293	2.122	2.010–2.239


**Figure 1:**
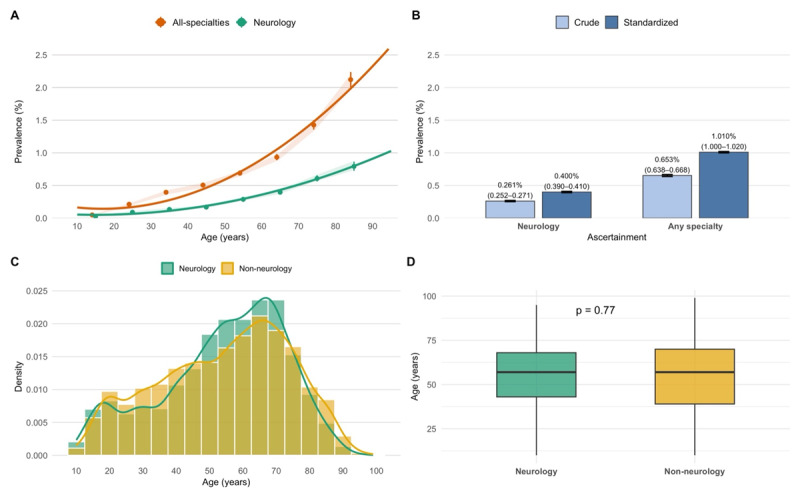
**Age-Specific and Standardized Prevalence of Essential Tremor by Ascertainment Strategy. (A)** Age-specific crude prevalence of essential tremor by ascertainment method. Points represent observed prevalence with 95% confidence intervals; curves represent quadratic polynomial fits. **(B)** Crude and age-standardized prevalence estimates by ascertainment strategy. Error bars represent 95% confidence intervals; standardized estimates were derived using direct age standardization. **(C)** Distribution of age at diagnosis in the mutually exclusive neurology and non-neurology cohorts, shown as histograms with density overlays. **(D)** Age at diagnosis in the neurology and non-neurology cohorts, presented as boxplots; no significant difference was observed between groups (*p* = 0.77).

Figure 1 (Panels A–B) better reflects the impact of ascertainment strategy on both age-specific and standardized prevalence of ET and on the distribution of age at diagnosis. Age-specific prevalence increased progressively with age for both neurology and all-specialties ascertainment, with consistently higher estimates observed in the all-specialties group (Panel A). The disparity between approaches became more pronounced in older age groups, surpassing 2% among individuals aged 80 years or older under all-specialties ascertainment, compared to approximately 0.8% with neurology criteria. Age-standardization resulted in slightly higher overall prevalence estimates than crude values, with a more substantial increase in the all-specialties cohort (Panel B). In a secondary analysis comparing mutually exclusive neurology and non-neurology cohorts, the age distribution of ET diagnoses was similar between groups, with peak density occurring in the sixth to seventh decades of life (Panel C). No statistically significant difference in mean age at diagnosis was observed between the neurology and non-neurology cohorts (*p* = 0.77; Panel D). In the neurology cohort (n = 2,827), the median age was 57 years, interquartile range (IQR) was 43–68 years, mean age was 54.3 years, standard deviation (SD) 18.5 years, with a range from 10 to 95 years. In the non-neurology cohort (n = 7065–2827 = 4,238), the median was also 57 years (IQR 39–70), with a mean of 54.2 years (SD 19.6 years) and a range from 10 to 99 years. These findings suggest that, although the age structure at the time of ET diagnosis is consistent across ascertainment methods, the absolute prevalence is significantly influenced by the range of diagnostic sources.

Sex-stratified analyses demonstrated age-related patterns similar to those observed in the overall cohort, with a modest female predominance in age-specific prevalence that became more pronounced at older ages (Tables 2 and 3). For individuals aged 10 years or older, the neurology ascertainment resulted in a crude prevalence of 0.244% (95% CI 0.231–0.257) in males and 0.279% (95% CI 0.265–0.293) in females. The corresponding all-specialties estimates were 0.586% (95% CI 0.565–0.606) for males and 0.719% (95% CI 0.697–0.742) for females. The observed female excess increased in the 60–69 and 70 years or older groups under both ascertainment strategies, although differences remained modest, typically less than 0.15 percentage points. In the 80 years or older stratum, all-specialties prevalence remained higher in females, whereas neurology estimates showed a slight reversal.

**Table 2:** Age-specific and Age-threshold Prevalence of Essential Tremor by Ascertainment Strategy in Males.


AGE-SPECIFIC	MALES	NEUROLOGY			ALL-SPECIALTIES		
	
		CASES	PREVALENCE	95% CI	CASES	PREVALENCE	95% CI

**10–19**	73,685	25	0.034	0.022–0.050	37	0.050	0.035–0.069

**20–29**	90,077	82	0.091	0.072–0.113	182	0.202	0.174–0.234

**30–39**	73,451	100	0.136	0.111–0.166	285	0.388	0.344–0.436

**40–49**	68,665	102	0.149	0.121–0.180	317	0.462	0.412–0.515

**50–59**	81,057	212	0.262	0.228–0.299	493	0.608	0.556–0.664

**60–69**	77,971	297	0.381	0.339–0.427	672	0.862	0.798–0.929

**70–79**	48,238	294	0.609	0.542–0.683	685	1.420	1.316–1.530

**AGE-THRESHOLD**		–	–	–	–	–	–

**10+**	535,931	1305	0.244	0.231–0.257	3138	0.586	0.565–0.606

**20+**	462,246	1280	0.277	0.262–0.292	3101	0.671	0.648–0.695

**30+**	372,169	1198	0.322	0.304–0.341	2919	0.784	0.756–0.813

**40+**	298,718	1098	0.368	0.346–0.390	2634	0.882	0.849–0.916

**50+**	230,053	996	0.433	0.407–0.461	2317	1.007	0.967–1.049

**60+**	148,996	784	0.526	0.491–0.564	1824	1.224	1.170–1.281

**70+**	71,025	487	0.686	0.628–0.749	1152	1.622	1.532–1.718

**80+**	22,787	193	0.847	0.736–0.975	467	2.049	1.873–2.242


**Table 3:** Age-specific and Age-threshold Prevalence of Essential Tremor by Ascertainment Strategy in Females.


AGE-SPECIFIC	FEMALES	NEUROLOGY			ALL-SPECIALTIES		
	
		CASES	PREVALENCE	95% CI	CASES	PREVALENCE	95% CI

**10–19**	72,036	15	0.021	0.012–0.034	34	0.047	0.033–0.066

**20–29**	82,395	74	0.090	0.071–0.113	184	0.223	0.192–0.258

**30–39**	69,484	90	0.130	0.104–0.159	281	0.404	0.359–0.454

**40–49**	66,580	127	0.191	0.159–0.227	367	0.551	0.496–0.610

**50–59**	80,563	254	0.315	0.278–0.356	621	0.771	0.712–0.834

**60–69**	80,713	333	0.413	0.370–0.459	810	1.004	0.936–1.075

**70–79**	56,082	339	0.604	0.542–0.672	804	1.434	1.337–1.535

**AGE-THRESHOLD**		–	–	–	–	–	–

**10+**	546,003	1522	0.279	0.265–0.293	3927	0.719	0.697–0.742

**20+**	473,967	1507	0.318	0.302–0.334	3893	0.821	0.796–0.847

**30+**	391,572	1433	0.366	0.348–0.385	3709	0.947	0.917–0.978

**40+**	322,088	1343	0.417	0.395–0.440	3428	1.064	1.029–1.100

**50+**	255,508	1216	0.476	0.450–0.503	3061	1.198	1.157–1.241

**60+**	174,945	962	0.550	0.516–0.586	2440	1.395	1.341–1.451

**70+**	94,232	629	0.668	0.617–0.722	1630	1.730	1.648–1.815

**80+**	38,150	290	0.760	0.678–0.852	826	2.165	2.024–2.316


**Figure 2:**
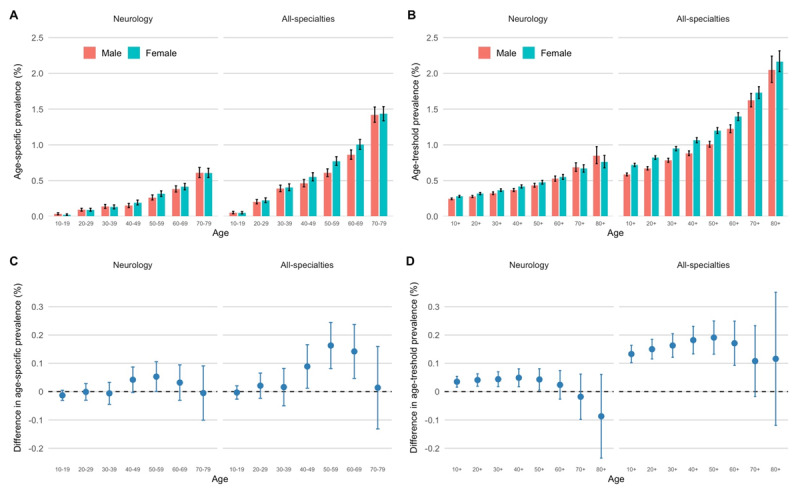
**Sex Differences in Essential Tremor Prevalence Across Age Groups and Ascertainment Strategies. (A)** Age-specific prevalence of essential tremor by sex, stratified by ascertainment method. **(B)** Prevalence of essential tremor by sex across increasing age-threshold groups. **(C)** Difference in age-specific prevalence between females and males (female – male) across age groups. **(D)** 95% confidence intervals for the differences in prevalence estimates between females and males across age threshold groups.

Figure 2 illustrates sex-specific prevalence of ET across age groups and ascertainment strategies. Crude prevalence increased steadily with advancing age in both males and females under neurology and all-specialties definitions, with some higher point estimates in females, particularly in older age (Panels A and B). This female predominance was more evident under all-specialties ascertainment, where prevalence exceeded 2% among females aged ≥80 years. Differences in age-specific prevalence estimates (female minus male) were small at younger ages and became more apparent with age, especially in the all-specialties cohort (Panel C). In nested analyses, the female-male difference remained positive for most age-threshold groups, but confidence intervals included zero in several strata (Panel D). The female predominance observed in all-specialties ascertainment was not evident in neurology-restricted analyses, suggesting that observed sex differences in ET prevalence may be influenced by the ascertainment strategy and may reflect differences in healthcare utilization or the capture of milder disease.

Overall, the approximately two-to-three-fold higher prevalence observed with all-specialties ascertainment compared with neurology-restricted ascertainment was consistent across age and sex strata, highlighting the substantial influence of case ascertainment strategy on EHR-based estimates of ET prevalence in NECP.

## Discussion

In this large population-based study leveraging EHR cases from GHS anchored to 2020 U.S. Census population counts of 1,081,934 individuals aged ≥10 years in NECP, the crude prevalence of ET was 0.261% (95% CI 0.252–0.271) under neurology ascertainment and 0.653% (95% CI 0.638–0.668) under all-specialties cohort. Corresponding age-standardized prevalence estimates were 0.40% (95% CI 0.39–0.41) and 1.01% (95% CI 1.00–1.02) respectively, reflecting an approximately 2.5-fold difference attributable to case definition breadth. Prevalence increased markedly with age under both approaches, rising from <0.05% in the 10–19-year stratum to 0.793% (neurology) and 2.122% (all-specialties) among those aged 80 years or older. Sex-stratified analyses revealed broadly parallel age gradients in males and females, with a modest female predominance evident particularly in older age groups (e.g., ≥60 years) clearer in the all-specialties ascertainment strategy. Notably, the age distribution at diagnosis remained comparable between neurology and non-neurology cohorts (p = 0.77), indicating that the ascertainment strategy primarily modulates case capture volume rather than the underlying demographic profile of diagnosed individuals. Taken together, these findings support a spectrum model of ET prevalence, ranging from specialist-confirmed disease to broader healthcare-recognized cases and, ultimately, to the full burden detected through population-based examination.

Early U.S. community-based studies, such as the Copiah County study [9], reported similar prevalence estimates of ET, approximately 0.4% among individuals aged ≥40 years. These lower estimates likely reflect restrictive diagnostic criteria that require long-standing, functionally significant tremor, thereby excluding milder or earlier-stage cases. In our study, the prevalence of ET among individuals aged ≥40 years was 0.393% (95% CI 0.378–0.409) using a neurology approach, supporting that stricter case definitions yield lower prevalence. Medical record-based investigations, such as the Rochester, Minnesota study by Rajput et al., reported age and sex-adjusted prevalence of ET of 0.306% [10]. This estimate closely aligns with our age-standardized neurology-only prevalence of 0.40% (95% CI 0.39–0.41), despite differences in population and time period.

Studies from Northern Manhattan by Louis et al. have reported substantially higher prevalence estimates of ET, ranging from approximately 4.0% to 5.5% among individuals aged ≥65 years, likely due to systematic population-based assessment and broader case capture, including mild and previously unrecognized cases [11,13]. In our cohort aged ≥60 years, prevalence was considerably lower at 0.539% (95% CI 0.514–0.565) using neurology ascertainment and 1.316% (95% CI 1.278–1.356) using all-specialties ascertainment, suggesting that a substantial proportion of cases in the community remain undiagnosed or unrecorded in routine clinical care. In a cohort of a retirement community with individuals 65 years or older, Khatter et al. conducted active screening and examination. They reported a high prevalence of tremor (43.5%), with ET identified in 20.5% of participants. Notably, this estimate included both symptomatic (11.8%) and asymptomatic cases, reflecting a broad definition that captured mild and previously unrecognized tremor [16]. In addition, the broader diagnostic definitions used in earlier community-based studies, which did not require prolonged disease duration and permitted inclusion of milder tremor syndromes, may further contribute to higher reported prevalence compared to more recent, stricter classification frameworks [1].

In a large multicenter cohort study conducted in the U.S., Louis et al. found that the prevalence of physician-diagnosed ET was 1.5% among individuals aged 65 and older [12]. This figure reflects only the cases that have been recognized within the healthcare system. It is significantly lower than estimates from community studies that utilize examinations, but it closely matches our estimates across all specialties. This suggests that even comprehensive EHR-based assessments primarily capture cases of ET that have been clinically recognized. In a community-based study using direct neurological examination, Louis et al. reported a crude prevalence of ET of 4.53%, with age-standardized estimates of 1.57% [14]. They demonstrated that most cases were previously undiagnosed. More recent administrative claims-based research, including the 2025 study by Lin et al., reported an age-standardized prevalence of diagnosed ET of 0.42%, closely mirroring the present neurology-restricted estimate of 0.40% [15].

The current findings thus occupy an intermediate position within this continuum, reconciling variability across the literature by demonstrating that neurology-restricted ascertainment approximates the lower bound of clinically recognized ET observed in record-linkage claims. This pattern underscores that EHR-derived prevalence reflects the spectrum of diagnosed disease within healthcare systems rather than the full population burden, which is likely underestimated, particularly among individuals with milder or unrecognized tremor. The observed differences suggest that a substantial proportion of ET cases are managed outside neurology, likely reflecting milder disease. This has implications for referral patterns, diagnostic recognition, and access to specialty care.

A central contribution of this analysis is the quantification of the influence of the ascertainment strategy within a single, well-defined regional population. The consistent approximately 2.5-fold elevation in prevalence under all-specialties versus neurology definitions across age and sex strata highlights that many ET cases are managed outside neurology, often with milder symptoms. Nevertheless, the comparable age-at-diagnosis distributions indicate that broader ascertainment did not systematically alter the age profile of the identified ET cases. Methodologically, our study advances and extends prior work by anchoring prevalence calculations to 2020 Census denominators for a precisely defined regional population and by integrating validated neurologist-assigned ET diagnoses with structured EHR elements to reduce potential misclassification arising from overlapping tremor syndromes.

The slight female predominance observed, though small in absolute magnitude and with overlapping confidence intervals in several strata, differs slightly from findings of some meta-analyses that report either no sex difference [8] or a slight male predominance in examination-based studies [26]. Potential explanations include differences in healthcare-seeking behavior. These findings merit cautious interpretation and further investigation rather than definitive attribution to biological differences.

Several limitations warrant consideration. First, reliance on EHR data inherently captures clinically recognized disease rather than true population prevalence, potentially underestimating the burden among individuals with mild, unrecognized, or unmanaged tremor who do not seek for care. Second, despite enhanced classification through the incorporation of clinical context and validation data showing favorable performance of neurologist-assigned diagnoses, residual misclassification remains possible, particularly in non-neurology cohort. Third, the single-health-system setting in NECP may limit generalizability to more diverse or urban populations with differing access, referral, or coding dynamics. Finally, unmeasured factors including provider variability and evolving diagnostic criteria over time could influence case ascertainment, especially in the all-specialties cohort.

Clinically and epidemiologically, these findings have several implications. They reinforce the strong association between the ET prevalence and age, with prevalence exceeding 1–2% in older age groups under broader case definitions, underscoring the need for heightened clinical awareness and appropriate resource planning in geriatric care. The sensitivity of prevalent estimates to case definition emphasizes the value of standardized, context-enriched EHR approaches for surveillance while also highlighting their distinction from examination-based methods, which may better approximate the true burden. More broadly, this study supports the utility of validated, population-anchored EHR analyses for large-scale epidemiologic research on movement disorders.

In conclusion, this census-linked EHR study shows that estimates of ET prevalence in NECP vary substantially by ascertainment strategy (0.40% neurology vs. 1.01% all-specialties after age standardization) while consistently exhibiting a strong age gradient. These findings support the hypothesis that reported ET prevalence varies systematically according to ascertainment strategy applied within the same population. Because case ascertainment relied on EHR data, the resulting estimates reflect healthcare-recognized and clinically diagnosed ET rather than the full burden of ET in the community. The lower prevalence observed compared to examination-based studies likely reflects individuals who have not sought medical care, have not received an ET diagnosis, or have not been captured within the healthcare system. Consequently, these findings should be interpreted as estimates of diagnosed ET prevalence rather than of population-wide ET prevalence.

## Generative AI

During the preparation of this work, the authors used generative artificial intelligence (GenAI) tools to assist with language editing, grammar refinement, clarity of expression, and code development. After using these tools, the authors carefully reviewed and edited the content as needed, verified all references and scientific statements, and took full responsibility for the accuracy and integrity of the published article.

## Data Availability

The aggregated data supporting the findings of this study are included in this published article and its supplementary materials. Individual-level data are not publicly available due to institutional restrictions on patient confidentiality. Still, they may be made available from the corresponding author upon reasonable request and with permission from Geisinger Medical Center.
